# Quality of life outcomes including neuropathy-associated scale from a phase II, multicenter, randomized trial of eribulin plus gemcitabine versus paclitaxel plus gemcitabine as first-line chemotherapy for HER2-negative metastatic breast cancer: Korean Cancer Study Group Trial (KCSG BR13-11)

**DOI:** 10.1186/s40880-019-0375-7

**Published:** 2019-05-28

**Authors:** Ji-Yeon Kim, Seri Park, Seock-Ah Im, Sung-Bae Kim, Joohyuk Sohn, Keun Seok Lee, Yee Soo Chae, Ki Hyeong Lee, Jee Hyun Kim, Young-Hyuck Im, Tae-Yong Kim, Kyung-Hun Lee, Jin-Hee Ahn, Gun Min Kim, In Hae Park, Soo Jung Lee, Hye Sook Han, Se Hyun Kim, Kyung Hae Jung, Yeon Hee Park, Korean Cancer Study Group

**Affiliations:** 1 Division of Hematology-Oncology, Department of Medicine, Samsung Medical Center, Sungkyunkwan University School of Medicine, 81 Irwon-ro, Gangnam-gu, Seoul, 06351 South Korea; 2 Department of Health Sciences and Technology, Samsung Advanced Institute for Health Sciences and Technology, Sungkyunkwan University, Seoul, 06351 South Korea; 3 Department of Internal Medicine, Seoul National University Hospital, Cancer Research Institute, College of Medicine, Seoul National University, Seoul, 03080 South Korea; 4 Department of Oncology, Asan Medical Center, University of Ulsan College of Medicine, Seoul, 05505 South Korea; 5 Division of Medical Oncology, Department of Internal Medicine, Yonsei University College of Medicine, Seoul, 03722 South Korea; 6 Center for Breast Cancer, National Cancer Center, Goyang, 10408 South Korea; 7 Department of Internal Medicine, Kyungpook National University Hospital, Daegu, 41944 South Korea; 8 Deparment of Internal Medicine, Chungbuk National University Hospital, Cheongju, 28644 South Korea; 9 Department of Internal Medicine, Seoul National University Bundang Hospital, Seoul National University College of Medicine, Seongnam, 13620 South Korea

**Keywords:** Metastatic breast cancer, Eribulin, Paclitaxel, Neuropathy, Quality of life, Functional assessment of cancer therapy-taxane questionnaires

## Abstract

**Background:**

A phase II clinical trial of the comparison between eribulin plus gemcitabine (EG) and paclitaxel plus gemcitabine (PG) as first-line chemotherapy for patients with metastatic breast cancer (MBC) found that the EG regimen was less neurotoxic, but was similar in efficacy to the PG regimen. In the present study, we analyzed functional assessment of cancer therapy-taxane (FACT-Taxane) questionnaires from patients in this clinical trial to determine their quality of life (QoL).

**Methods:**

QoL was assessed using the Korean version of the FACT-Taxane questionnaires. After baseline assessment, QoL was assessed every 2 cycles for 12 cycles and every 3 cycles thereafter. The linear mixed model was used to evaluate the difference in QoL between the EG and PG arms.

**Results:**

Of the 118 enrolled patients, 117 responded to the FACT-Taxane questionnaires at baseline, 1 in the PG arm did not. Baseline QoL scores were not different between the EG and PG arms. During treatment, taxane subscale scores were significantly higher in the PG arm than in the EG arm after 2–13 cycles of chemotherapy (all *P* < 0.05), except for the 11th cycle. Neuropathy-specific analysis showed that patients in the PG arm had earlier and more severe neuropathic symptoms than those in the EG arm (*P *< 0.001).

**Conclusions:**

In our QoL analysis, the EG regimen delayed and decreased neuropathy as compared with the PG regimen. Therefore, eribulin would be a reasonable substitute for paclitaxel as first-line chemotherapy for MBC.

**Electronic supplementary material:**

The online version of this article (10.1186/s40880-019-0375-7) contains supplementary material, which is available to authorized users.

## Background

The goal of palliative chemotherapy is to prolong survival without cancer-related symptoms. Therefore, patient-reported quality of life (QoL), as like as survival outcome, is an important endpoint in evaluating treatment efficacy in metastatic breast cancer (MBC) patients [1]. Moreover, QoL of MBC patients was an independent prognostic marker [2].

Paclitaxel is an effective chemotherapeutic agent and an essential part of treatment for MBC patients [3, 4]. However, it leads to neuropathy in most patients, and severe neuropathy is a main reason for discontinuation of treatment. Previous studies of adjuvant taxane chemotherapy presented that about 20%–30% of patient had persistent peripheral neuropathy after chemotherapy discontinuation [5, 6]. In metastatic setting, paclitaxel plus gemcitabine (PG regimen) followed by maintenance gemcitabine prolonged progression-free survival and overall survival, but approximately 90% of patients experienced grade 2–3 neuropathy [7]. Moreover, chemotherapy-induced peripheral neuropathy had a negative association with QoL of patients [8]. Therefore, prevention of neuropathy may improve QoL of patients with breast cancer during treatment.

Eribulin, a non-taxane microtubule inhibitor, is an effective therapeutic agent for MBC patients [9]. It induced neuropathy in about 30% of patients, which was significantly lower than the rate of paclitaxel-induced neuropathy [10]. Taking this into account, we conducted a phase II, multicenter, randomized, open-labeled clinical trial comparing eribulin plus gemcitabine (EG regimen) with PG regimen as first-line chemotherapy for patients with human epidermal growth factor receptor 2 (HER2)-negative MBC (the Korean Cancer Study Group [KCSG] BR13-11, Clinicaltrials.gov, NCT02263495) [11]. This clinical trial determined that treatment with EG resulted in similar outcomes as treatment with PG, but was less neurotoxic. Other toxicities of both treatments were reported at comparable levels.

In the present study, we analyzed QoL of participating patients in this clinical trial using the functional assessment of cancer therapy-taxane (FACT-Taxane) questionnaires. Especially, we focused on chemotherapy-induced peripheral neuropathy in the two therapeutic arms.

## Patients and methods

### Patient selection

Women with histologically confirmed HER2-negative MBC and with no history of chemotherapy for metastatic disease were eligible for KCSG BR13-11 trial [11]. Patients were eligible for the study if at least 12 months had passed since the completion of prior chemotherapy, even if they had received an anthracycline- or taxane-containing regimen as neoadjuvant or adjuvant therapy. Patients with grade 2 or higher peripheral neuropathy were excluded.

### Chemotherapy

Patients were randomly assigned, in a 1:1 ratio, to either the EG or PG treatment arm using interactive web response system (IWRS) [11]. EG chemotherapy comprised intravenous administration of 1.0 mg/m^2^ eribulin and 30-min intravenous infusion of 1000 mg/m^2^ gemcitabine on days 1 and 8 every 3 weeks. PG chemotherapy comprised intravenous administration of 175 mg/m^2^ paclitaxel on day 1 with 30-min intravenous infusion of 1250 mg/m^2^ gemcitabine on days 1 and 8 every 3 weeks.

The KCSG BR13-11 trial was conducted in full accordance with the guidelines for Good Clinical Practice and the Declaration of Helsinki, and was approved by the institutional ethics committees of each hospital and the KCSG institutional review board. The ClinicalTrials.gov identifier number was NCT02263495. Written informed consent for publication of their clinical details was obtained from all patients.

Neurotoxicity was assessed at the end of each cycle using National Cancer Institute Common Toxicity Criteria (NCI-CTC), version 4.0.

### QoL assessment

QoL was assessed using the Korean version of the FACT-Taxane questionnaires, which comprise the FACT-general (FACT-G) and taxane subscale questionnaires [12]. FACT-G contains four subscales measuring physical well-being (PWB), social well-being (SWB), emotional well-being (EWB), and functional well-being (FWB). The four subscales contain 7, 7, 6, and 7 items, respectively. The taxane subscale is a 16-item self-reporting questionnaire that focuses on patient-reported neuropathic symptoms and concerns. The FACT questionnaires use a 5-point response scale: 0 indicates not at all; 1, a little bit; 2, somewhat; 3, quite a bit; and 4, very much. The ranges of scores are 0–108 for FACT-G and 0–64 for taxane subscale. In FACT-G, the ranges of possible scores for the physical, social, emotional, and functional subscales are 0–28, 0–28, 0–24, and 0–28, respectively.

Patients were asked to complete the first QoL assessment before randomization. QoL was assessed every 2 chemotherapy cycles for 12 cycles and every 3 cycles thereafter. Assessment continued until the treatment was terminated due to disease progression, withdrawal, and intolerable adverse events.

### Statistical analyses

Differences in patient reports between the two treatment arms were calculated using the Student *t* test. QoL scores (mean and standard deviation) were calculated at baseline and each assessed point for the overall scale (the sum of all subscales) as well as for the PWB, SWB, EWB, FWB, and taxane subscales. The linear mixed model was used to examine the QoL difference between the two treatment arms. The time to neuropathy onset was reported using the reverse Kaplan–Meier method. Relative dose intensity was defined as the amount of drug administered per unit time that expressed as a percentage of the planned dose or standard regimen. A two-sided *P* value < 0.05 was considered significant. SPSS Statistics ver. 21 (IBM Co., Armonk, NY, USA) was used for analysis of all data.

## Results

### Patient characteristics

Between December 2014 and March 2016, a total of 118 patients were enrolled and randomly assigned to either the EG arm or the PG arm at a ratio of 1:1. The median number of chemotherapy cycles was 10 (range 3–40) in the EG arm and 8 (range 2–34) in the PG arm. Of the 118 patients, 1 in the PG arm did not respond to the FACT-Taxane questionnaires at baseline, and 7 in the EG arm did not respond at the end of treatment. In terms of fidelity of questionnaire, no incomplete responses were recorded. Overall, the complete response rate was 99.0%. Additional file 1: Table S1 shows the number of patients enrolled in this study and the number of those who responded to questionnaires in each arm.

### Changes in general QoL during treatment

**Fig. 1:**
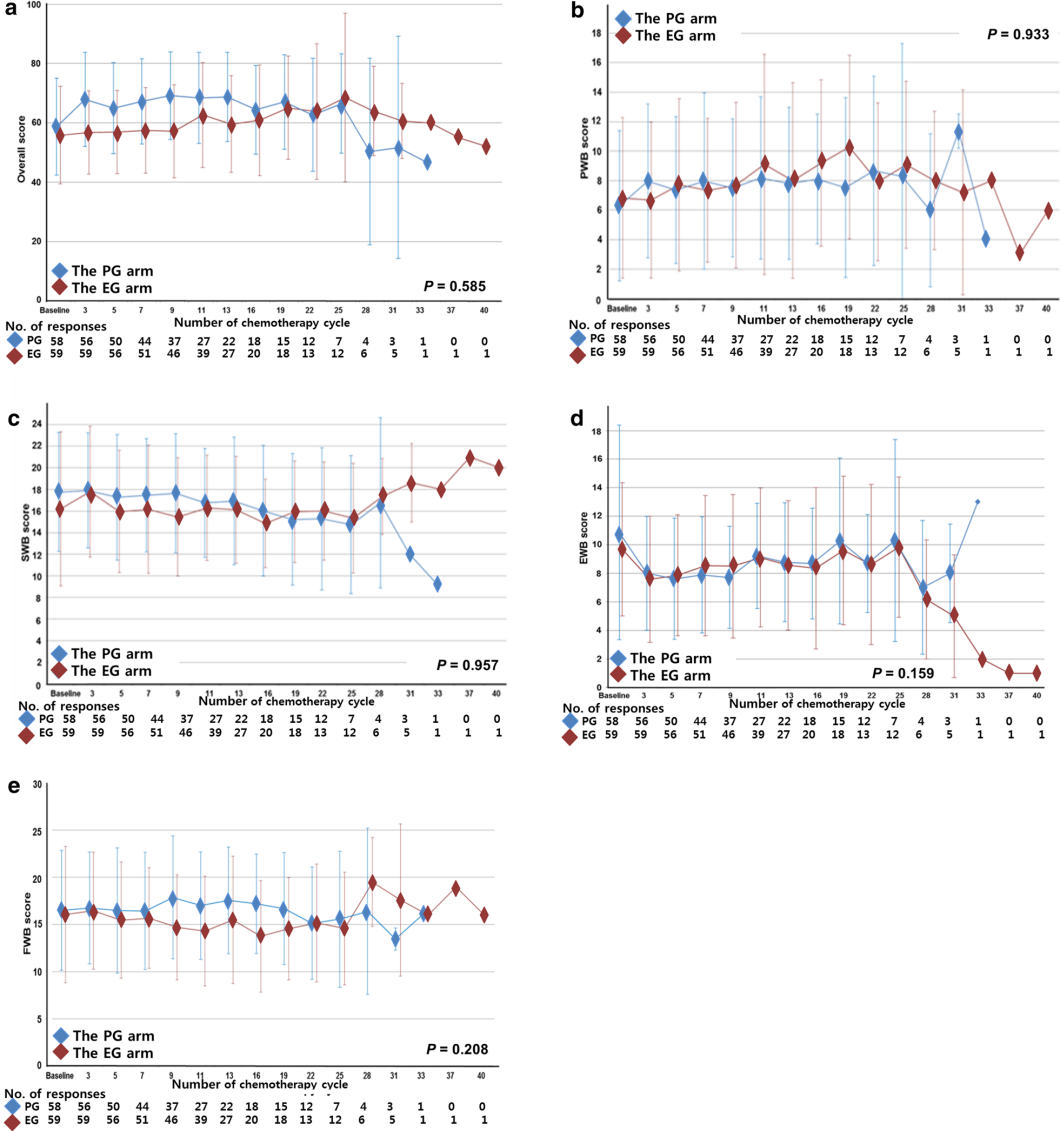
Functional assessment of cancer therapy (FACT)-General for the quality of life of patients with human epidermal growth factor receptor 2 (HER2)-negative metastatic breast cancer (MBC) in the eribulin plus gemcitabine (EG) and paclitaxel plus gemcitabine (PG) arms. **a** Overall scores; **b** physical well-being (PWB) scores; **c** social well-being (SWB) scores; **d** emotional well-being (EWB) scores; **e** functional well-being (FWB) scores. All data are presented as mean values, with bars indicate standard deviation

We assessed treatment-related changes in FACT-G scores between the two arms. No differences in PWB, SWB, EWB, FWB, and overall scores at baseline and during treatment were observed (all *P* > 0.05) (Fig. 1).

### Changes in taxane-associated QoL during treatment

**Fig. 2:**
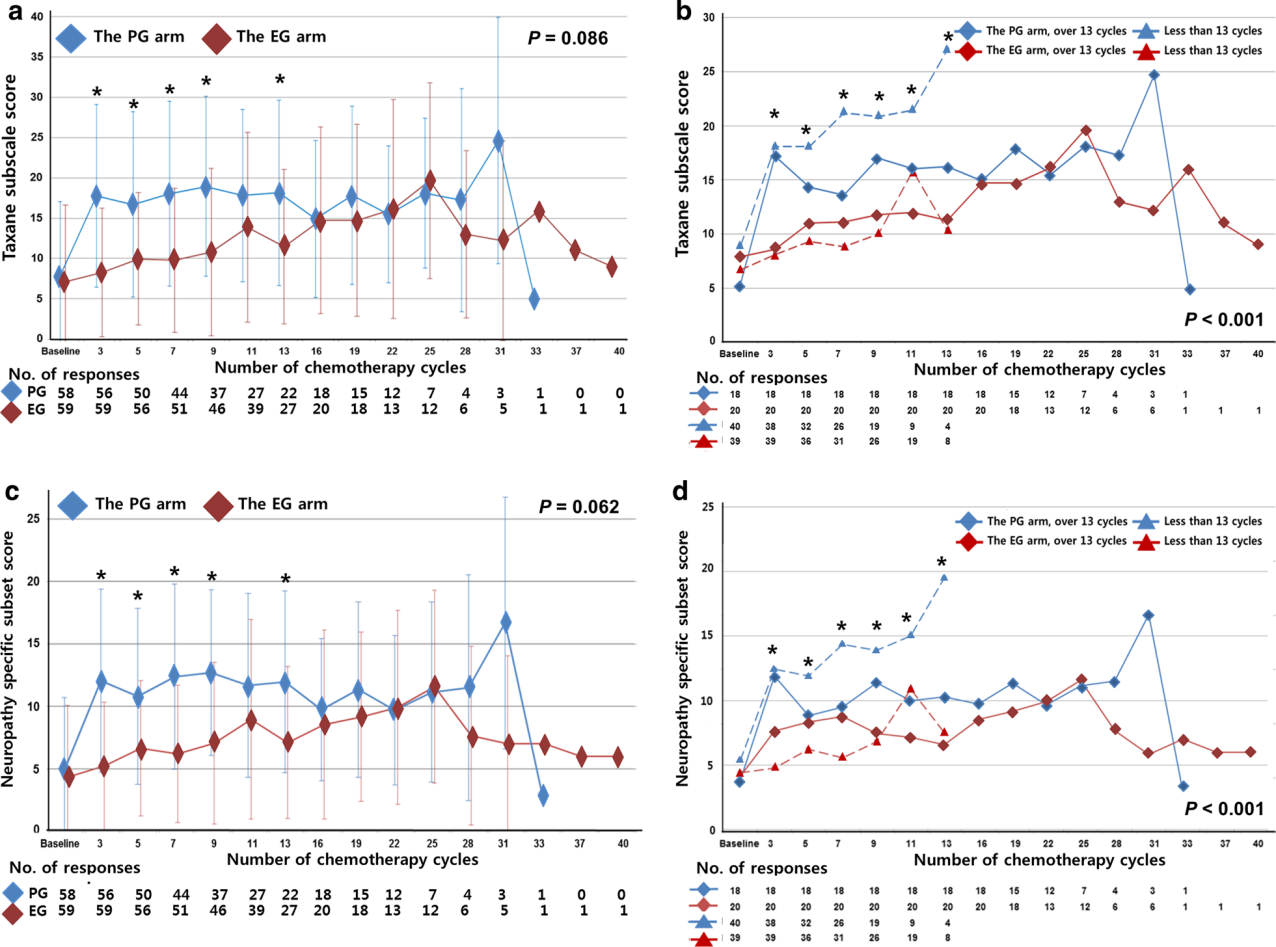
Taxane subscale scores for patients with HER2-negative MBC in the EG and PG arms. **a** Taxane subscale scores for the EG and PG arms; **b** taxane subscale scores for patients treated with less than 13 cycles or over 13 cycles of the EG or PG regimen; **c** neuropathy-specific subset scores for the EG and PG arms; **d** neuropathy-specific subset scores for patients treated with less than 13 cycles or over 13 cycles of the EG or PG regimen. All data are presented as mean values, with bars indicate standard deviation

The baseline taxane subscale score did not differ between the EG and PG arms (*P* = 0.684). During treatment, the taxane subscale score was found to increase earlier in the PG arm than in the EG arm. Overall, taxane subscale scores were higher in the PG arm than in the EG arm, but with no significant differences (*P* = 0.086) (Additional file 1: Table S2). During the first 13 cycles of treatment, the taxane subscale score was significantly higher in the PG arm than in the EG arm (all *P* < 0.05), except for the assessment at the 11th cycle; after then, no significant difference between the two arms was observed (Fig. 2a).

We performed subgroup analysis according to cycles of treatment. Because relative dose intensities of eribulin and paclitaxel were crossed at the 11th cycle of treatment (Additional file 2: Fig. S1), we chose the 13th cycle as cut-off value of treatment according to the point of QoL assessment and divided each arm into two subgroups: patients treated with less than 13 cycles and over 13 cycles. Patients treated with less than 13 cycles of PG had the highest taxane subscale score among the four subgroups (*P *< 0.001) (Fig. 2b and Additional file 1: Table S3). Compared between the two subgroups of less than 13 cycles, the mean taxane subscale score in the PG arm was almost twice as high as that in the EG arm (*P *< 0.001). However, the mean score did not differ between the two subgroups of over 13 cycles (*P* = 0.498) (Additional file 1: Table S3).

Of the 16 items of the taxane subscale, nine were neuropathy-specific questions. We further analyzed the intensity of chemotherapy-induced peripheral neuropathy. The trend of changes in neuropathy-specific subset scores was similar to that of taxane subscale scores for both arms and for the four subgroups (Fig. 2c, d and Additional file 1: Table S3).

### Peripheral neuropathy according to treatment

**Fig. 3:**
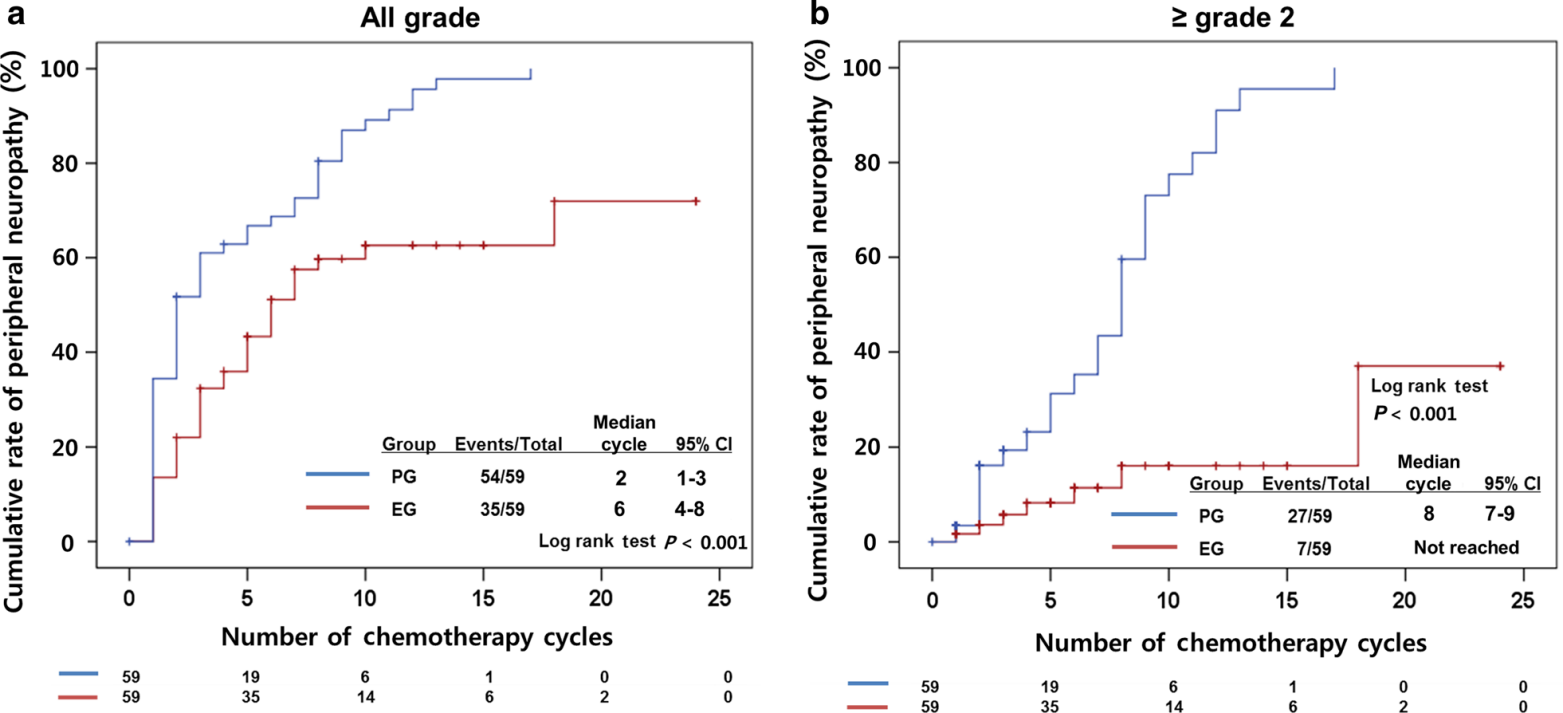
Cumulative rates of peripheral neuropathy of **a** all grades and of **b** grade 2 or higher

The cumulative rate of peripheral neuropathy was analyzed. Of the 118 patients, 89 experienced neuropathy: 54 in the PG arm and 35 in the EG arm (Fig. 3a). Moreover, patients in the PG arm had earlier development after a median of 2 cycles as compared with those in the EG arm after a median of 6 cycles (*P* < 0.001).

Thirty-four patients had peripheral neuropathy of grade 2 or higher: 27 in the PG arm and seven in the EG arm (Fig. 3b). The median time to development of peripheral neuropathy of grade 2 or higher was after 8 cycles in the PG arm and was not reached in the EG arm (*P* < 0.001).

Due to neuropathy, 16 (27.1%) patients in the PG arm and 3 (5.1%) in the EG arm required sequential dose modification. Fifteen of the 16 patients in the PG arm had dose reduction before completing 13 cycles of treatment, whereas the 3 EG patients in the EG arm did so at the 9th, 19th, and 21st cycles. The PG treatment was discontinued in 40 patients due to disease progression (55.0%), discretion of physician and patients (30.0%), adverse events (7.5%), and other reasons (7.5%) during the first 13 cycles of treatment. Thirty-five patients stopped EG treatment because of disease progression (85.7%), discretion (5.7%), adverse events (5.7%), and other reasons (2.9%). Actually, the relative dose intensity of eribulin was slightly decreased, whereas that of paclitaxel decreased at every cycle of treatment (Additional file 2: Fig. S1).

## Discussion

In our analysis of QoL, we found that EG chemotherapy delayed and decreased taxane-induced peripheral neuropathy as compared with PG chemotherapy. Although no significant difference was observed between the two arms during entire duration of treatment, the subgroup of patients who received less than 13 cycles of PG treatment had significantly more taxane-induced peripheral neuropathy than other subgroups.

These results were compatible with the data of physician-reported peripheral neuropathy. The events of peripheral neuropathy increased rapidly in PG arm till 13th cycles and the difference between EG and PG arms were expanded. Neuropathy occurred in relatively early period of treatment, and some patients withdrew their treatment due to neuropathy. Patient without neuropathy continued their treatment, and therefore there were no significant differences in QoL scores between the EG and PG arms after the 13th cycle. In addition, some patients in the EG arm developed neuropathy during later cycles, but many patients reported low neuropathy-specific subset scores.

Patient-reported QoL outcomes reflect quality of palliative care for cancer patients. QoL reporting was an independent prognostic marker, therefore many clinical trials for cancer patients analyzed patient-reported QoL data as a secondary endpoint [13]. Moreover, patient-reporting outcome measures have been considered as a routine practice nowadays [14]. Although our QoL analysis showed that there was little difference between patient- and physician-reported neuropathy, the analysis of patient-reported QoL data is needed for the prediction of prognosis.

In the present study, we found no significant differences in PWB, EWB, SWB, FWB, and overall scores between the EG and PG arms. Similarly, previous studies showed that taxane-based chemotherapy did not affect QoL scores assessed using the FACT-G questionnaire and other QoL questionnaires without taxane-specific questions [15–17].

Paclitaxel-induced peripheral neuropathy is related to high cumulative dose and high dose per cycle [18, 19]. In the present study, about 90% of patients treated with paclitaxel reported neuropathic symptoms. This is consistent with reported data from a previous PG maintenance study, which showed that nearly 90% of neuropathies occurred after 6 cycles of PG treatment [7]. In spite of the high rate of neuropathy, there are no definite preventive or treatment measures of neuropathy [20]. The American Society of Clinical Oncology (ASCO) guidelines for prevention and management of chemotherapy-induced peripheral neuropathy indicate that there are no agents for neuropathy prevention [21]. Duroxetine is recommended, but neuropathic pain still existed in half of patients after duroxetine treatment [22]. Eribulin also induces peripheral neuropathy, but with lower rate and grade as compared with those induced by taxane. EMBRACE, a phase III clinical trial of eribulin monotherapy, showed that 35% of eribulin-treated patients reported peripheral neuropathy, but only 8.2% were in grade 3–4 [10]. It was also reported that eribulin-induced neuropathic pain was alleviated in most patients within a short period [23]. Therefore, for patients at risk for paclitaxel-induced neuropathy [24], eribulin might be a good substitute.

The limitation of the present study was that this phase II clinical trial had a relatively small sample size. To confirm our results, a large-scale phase III clinical trial of comparison between eribulin and paclitaxel would be warranted.

## Conclusions

In the present QoL study, EG treatment decreased taxane-related adverse events, especially neuropathy, as compared with PG treatment. Accordingly, eribulin would be a reasonable substitute for paclitaxel as first-line chemotherapy for MBC.

## Additional files

**Additional file 1: Table S1.** Number of questionnaire responses at each point of assessment. **Table S2.** Comparison of FACT-Taxane scores of the EG and PG groups during treatment. **Table S3.** Comparison of Taxane-associated scores of the EG and PG groups during treatment.

**Additional file 2: Figure S1.** Relative dose intensities of eribulin and paclitaxel on each cycle. The red lines indicate trends of both arms.

## Data Availability

All data generated or analyzed during this study are included in this published article and its additional files.

## Abbreviations

QoL: quality of life
EG: eribulin plus gemcitabine
PG: paclitaxel plus gemcitabine
FACT-Taxane: functional assessment of cancer therapy-taxane
MBC: metastatic breast cancer
HER2: human epidermal growth factor receptor 2
KCSG: Korean Cancer Study Group
FACT-G: functional assessment of cancer therapy-general
PWB: physical well-being
SWB: social well-being
EWB: emotional well-being
FWB: functional well-being

## References

[1] Montazeri A (2008). Health-related quality of life in breast cancer patients: a bibliographic review of the literature from 1974 to 2007. J Exp Clin Cancer Res..

[2] Coates A, Gebski V, Signorini D, Murray P, McNeil D, Byrne M (1992). Prognostic value of quality-of-life scores during chemotherapy for advanced breast cancer. Australian New Zealand Breast Cancer Trials Group. J Clin Oncol.

[3] Albain KS, Nag SM, Calderillo-Ruiz G, Jordaan JP, Llombart AC, Pluzanska A (2008). Gemcitabine plus Paclitaxel versus Paclitaxel monotherapy in patients with metastatic breast cancer and prior anthracycline treatment. J Clin Oncol.

[4] Sledge GW, Neuberg D, Bernardo P, Ingle JN, Martino S, Rowinsky EK (2003). Phase III trial of doxorubicin, paclitaxel, and the combination of doxorubicin and paclitaxel as front-line chemotherapy for metastatic breast cancer: an intergroup trial (E1193). J Clin Oncol.

[5] Sparano JA, Wang M, Martino S, Jones V, Perez EA, Saphner T (2008). Weekly paclitaxel in the adjuvant treatment of breast cancer. N Engl J Med.

[6] Hershman DL, Unger JM, Crew KD, Minasian LM, Awad D, Moinpour CM (2013). Randomized double-blind placebo-controlled trial of acetyl-l-carnitine for the prevention of taxane-induced neuropathy in women undergoing adjuvant breast cancer therapy. J Clin Oncol.

[7] Park YH, Jung KH, Im SA, Sohn JH, Ro J, Ahn JH (2013). Phase III, multicenter, randomized trial of maintenance chemotherapy versus observation in patients with metastatic breast cancer after achieving disease control with six cycles of gemcitabine plus paclitaxel as first-line chemotherapy: KCSG-BR07-02. J Clin Oncol.

[8] Mols F, Beijers T, Vreugdenhil G, van de Poll-Franse L (2014). Chemotherapy-induced peripheral neuropathy and its association with quality of life: a systematic review. Support Care Cancer.

[9] Pivot X, Marme F, Koenigsberg R, Guo M, Berrak E, Wolfer A (2016). Pooled analyses of eribulin in metastatic breast cancer patients with at least one prior chemotherapy. Ann Oncol.

[10] Cortes J, O’Shaughnessy J, Loesch D, Blum JL, Vahdat LT, Petrakova K (2011). Eribulin monotherapy versus treatment of physician’s choice in patients with metastatic breast cancer (EMBRACE): a phase 3 open-label randomised study. Lancet.

[11] Park YH, Im SA, Kim SB, Sohn JH, Lee KS, Chae YS (2017). Phase II, multicentre, randomised trial of eribulin plus gemcitabine versus paclitaxel plus gemcitabine as first-line chemotherapy in patients with HER2-negative metastatic breast cancer. Eur J Cancer.

[12] Cella D, Peterman A, Hudgens S, Webster K, Socinski MA (2003). Measuring the side effects of taxane therapy in oncology: the functional assessment of cancer therapy-taxane (FACT-taxane). Cancer.

[13] Takada K, Kashiwagi S, Fukui Y, Goto W, Asano Y, Morisaki T (2019). Prognostic value of quality-of-life scores in patients with breast cancer undergoing preoperative chemotherapy. BJS Open..

[14] Howell D, Molloy S, Wilkinson K, Green E, Orchard K, Wang K (2015). Patient-reported outcomes in routine cancer clinical practice: a scoping review of use, impact on health outcomes, and implementation factors. Ann Oncol.

[15] Kawahara M, Tada H, Tokoro A, Teramukai S, Origasa H, Kubota K (2011). Quality-of-life evaluation for advanced non-small-cell lung cancer: a comparison between vinorelbine plus gemcitabine followed by docetaxel versus paclitaxel plus carboplatin regimens in a randomized trial: Japan Multinational Trial Organization LC00-03 (BRI LC03-01). BMC Cancer..

[16] Morgans AK, Chen YH, Sweeney CJ, Jarrard DF, Plimack ER, Gartrell BA (2018). Quality of life during treatment with chemohormonal therapy: analysis of E3805 chemohormonal androgen ablation randomized trial in prostate cancer. J Clin Oncol.

[17] Shimozuma K, Ohashi Y, Takeuchi A, Aranishi T, Morita S, Kuroi K (2012). Taxane-induced peripheral neuropathy and health-related quality of life in postoperative breast cancer patients undergoing adjuvant chemotherapy: N-SAS BC 02, a randomized clinical trial. Support Care Cancer.

[18] Postma TJ, Vermorken JB, Liefting AJ, Pinedo HM, Heimans JJ (1995). Paclitaxel-induced neuropathy. Ann Oncol.

[19] Lee JJ, Swain SM (2006). Peripheral neuropathy induced by microtubule-stabilizing agents. J Clin Oncol.

[20] Wolf S, Barton D, Kottschade L, Grothey A, Loprinzi C (2008). Chemotherapy-induced peripheral neuropathy: prevention and treatment strategies. Eur J Cancer.

[21] Hershman DL, Lacchetti C, Dworkin RH, Lavoie Smith EM, Bleeker J, Cavaletti G (2014). Prevention and management of chemotherapy-induced peripheral neuropathy in survivors of adult cancers: American Society of Clinical Oncology clinical practice guideline. J Clin Oncol.

[22] Smith EM, Pang H, Cirrincione C, Fleishman S, Paskett ED, Ahles T (2013). Effect of duloxetine on pain, function, and quality of life among patients with chemotherapy-induced painful peripheral neuropathy: a randomized clinical trial. JAMA.

[23] Peter K, Martin O, Yi H, McCutcheon S, Vahdat L (2014). Peripheral neuropathy (PN) in patients (pts) with metastatic breast cancer treated with eribulin: resolution and association with efficacy. J Clin Oncol..

[24] Song SJ, Min J, Suh SY, Jung SH, Hahn HJ, Im SA (2017). Incidence of taxane-induced peripheral neuropathy receiving treatment and prescription patterns in patients with breast cancer. Support Care Cancer.

