# Supplementary material for: Quality of life outcomes including neuropathy-associated scale from a phase II, multicenter, randomized trial of eribulin plus gemcitabine versus paclitaxel plus gemcitabine as first-line chemotherapy for HER2-negative metastatic breast cancer: Korean Cancer Study Group Trial (KCSG BR13-11)
Source: Cancer Commun (Lond). 2019 May 28;39:29. doi: 10.1186/s40880-019-0375-7 (PMC6540535; doi:10.1186/s40880-019-0375-7)
Supplement: Supplementary file 1 — Additional file 1: Table S1. Number of questionnaire responses at each point of assessment. Table S2. Comparison of FACT-Taxane scores of the EG and PG groups during treatment. Table S3. Comparison of Taxane-associated scores of the EG and PG groups during treatment. [file 40880_2019_375_MOESM1_ESM.docx]

**Table S1 Number of questionnaire responses at each point of assessment**

| Chemotherapy cycle | EG (*n* =59) | PG (*n* =59) |
| --- | --- | --- |
| Baseline | 59 (100%) | 58 (98.3%) |
| 3^rd^ | 59 (100%) | 57 (100%) |
| 5^th^ | 56 (98.2%) | 50 (100%) |
| 7^th^ | 51 (98.1%) | 44 (100%) |
| 9^th^ | 46 (100%) | 37 (100%) |
| 11^th^ | 39 (97.5%) | 27 (100%) |
| 13^th^ | 27 (96.4%) | 22 (100%) |
| 16^th^ | 20 (90.1%) | 18 (100%) |
| 19^th^ | 18 (94.7%) | 15 (100%) |
| 22^nd^ | 13 (100%) | 12 (100%) |
| 25^th^ | 12 (100%) | 7 (100%) |
| 28^th^ | 6 (100%) | 4 (100%) |
| 31^st^ | 5 (100%) | 3 (100%) |
| 34^th^ | 1 (100%) | 1 (100%) |
| 37^th^ | 1 (100%) | 0 |
| 40^th^ | 1 (100%) | 0 |

EG, eribulin plus gemcitabine; PG, paclitaxel plus gemcitabine.

All data are presented as the number of responses with response rate in parentheses.

**Table S2 Comparison of FACT-Taxane scores of the EG and PG groups during treatment**

| **FACT-Taxane score** | Estimate | Standard error | df | *t* | *P* | 95% CI |
| --- | --- | --- | --- | --- | --- | --- |
| PWB score |  |  |  |  |  |  |
| Intercept | 6.377 | 0.696 | 191.049 | 9.168 | < 0.001 | 5.005, 7.749 |
| PG (ref. EG) | 0.012 | 0.138 | 148.655 | 0.084 | 0.933 | -0.260, 0.284 |
| SWB score |  |  |  |  |  |  |
| Intercept | 16.871 | 0.685 | 166.950 | 24.625 | < 0.001 | 15.519, 18.224 |
| PG (ref. EG) | -0.006 | 0.112 | 178.917 | -0.054 | 0.957 | -0.227, 0.215 |
| EWB score |  |  |  |  |  |  |
| Intercept | 8.510 | 0.581 | 193.571 | 14.649 | < 0.001 | 7.364, 9.656 |
| PG (ref. EG) | -0.166 | 0.117 | 140.070 | -1.415 | 0.159 | -0.399, 0.066 |
| FWB score |  |  |  |  |  |  |
| Intercept | 16.484 | 0.757 | 169.561 | 21.786 | < 0.001 | 14.990, 17.977 |
| PG (ref. EG) | 0.162 | 0.129 | 147.608 | 1.264 | 0.208 | -0.092, 0.416 |
| Taxane subscale score |  |  |  |  |  |  |
| Intercept | 6.554 | 1.298 | 184.993 | 5.051 | < 0.001 | 3.993, 9.114 |
| PG (ref. EG) | 0.416 | 0.241 | 144.813 | 1.726 | 0.086 | -0.060, 0.892 |
| Neuropathy subset score |  |  |  |  |  |  |
| Intercept | 4.313 | 0.827 | 193.197 | 5.213 | < 0.001 | 2.681, 5.945 |
| PG (ref. EG) | 0.305 | 0.162 | 150.772 | 1.883 | 0.062 | -0.150, 0.624 |
| Overall score |  |  |  |  |  |  |
| Intercept | 55.718 | 2.082 | 211.505 | 26.767 | < 0.001 | 51.614, 59.821 |
| PG (Ref. EG) | 0.252 | 0.461 | 158.735 | 0.547 | 0.585 | -0.658, 1.162 |

*CI* Confidence Interval, *PWB* Physical well-being, *SWB* Social well-being, *EWB* Emotional well-being, *FWB* Functional well-being, *PG* Paclitaxel plus gemcitabine, *EG* Eribulin plus gemcitabine

**Table S3. Comparison of Taxane-associated scores of the EG and PG groups during treatment**

| **Taxane-associated score** | Estimate | Standard error | df | *t* | | *P* | 95% CI |
| --- | --- | --- | --- | --- | --- | --- | --- |
| Four-subgroup analysis (ref. EG over 13 cycles) | | | | | | | |
| Intercept | 7.450 | 2.110 | 162.384 | | 3.554 | < 0.001 | 3.332, 11.667 |
| PG less than 13 cycles | 2.062 | 0.438 | 237.308 | | 4.705 | < 0.001 | 1.199. 2.925 |
| EG less than 13 cycles | 0.410 | 0.381 | 197.960 | | 1.077 | 0.283 | -0.341, 1.162 |
| PG over 13 cycles | 0.221 | 0.270 | 117.557 | | 0.820 | 0.414 | -0.313, 0.756 |
| Two-subgroup analysis 1 (ref. EG less than 13 cycles) | | | | | | |  |
| Intercept | 5.322 | 1.787 | 134.697 | | 2.979 | 0.003 | 1.789, 8.856 |
| PG less than 13 cycles | 1.755 | 0.573 | 83.144 | | 3.066 | 0.003 | 0.617, 2.894 |
| Two-subgroup analysis 2 (ref. EG over 13 cycles) | | | | | | |  |
| Intercept | 7.423 | 2.156 | 56.046 | | 3.443 | 0.001 | 3.104, 11.741 |
| PG over 13 cycles | 0.173 | 0.253 | 91.668 | | 0.681 | 0.498 | -0.331, 0.676 |

*CI* Confidence Interval, *PWB* Physical well-being, *SWB* Social well-being, *EWB* Emotional well-being, *FWB* Functional well-being, *PG* Paclitaxel plus gemcitabine, *EG* Eribulin plus gemcitabine
