# Supplementary figures and images for: Quality of life outcomes including neuropathy-associated scale from a phase II, multicenter, randomized trial of eribulin plus gemcitabine versus paclitaxel plus gemcitabine as first-line chemotherapy for HER2-negative metastatic breast cancer: Korean Cancer Study Group Trial (KCSG BR13-11)
Source: Cancer Commun (Lond). 2019 May 28;39:29. doi: 10.1186/s40880-019-0375-7 (PMC6540535; doi:10.1186/s40880-019-0375-7)

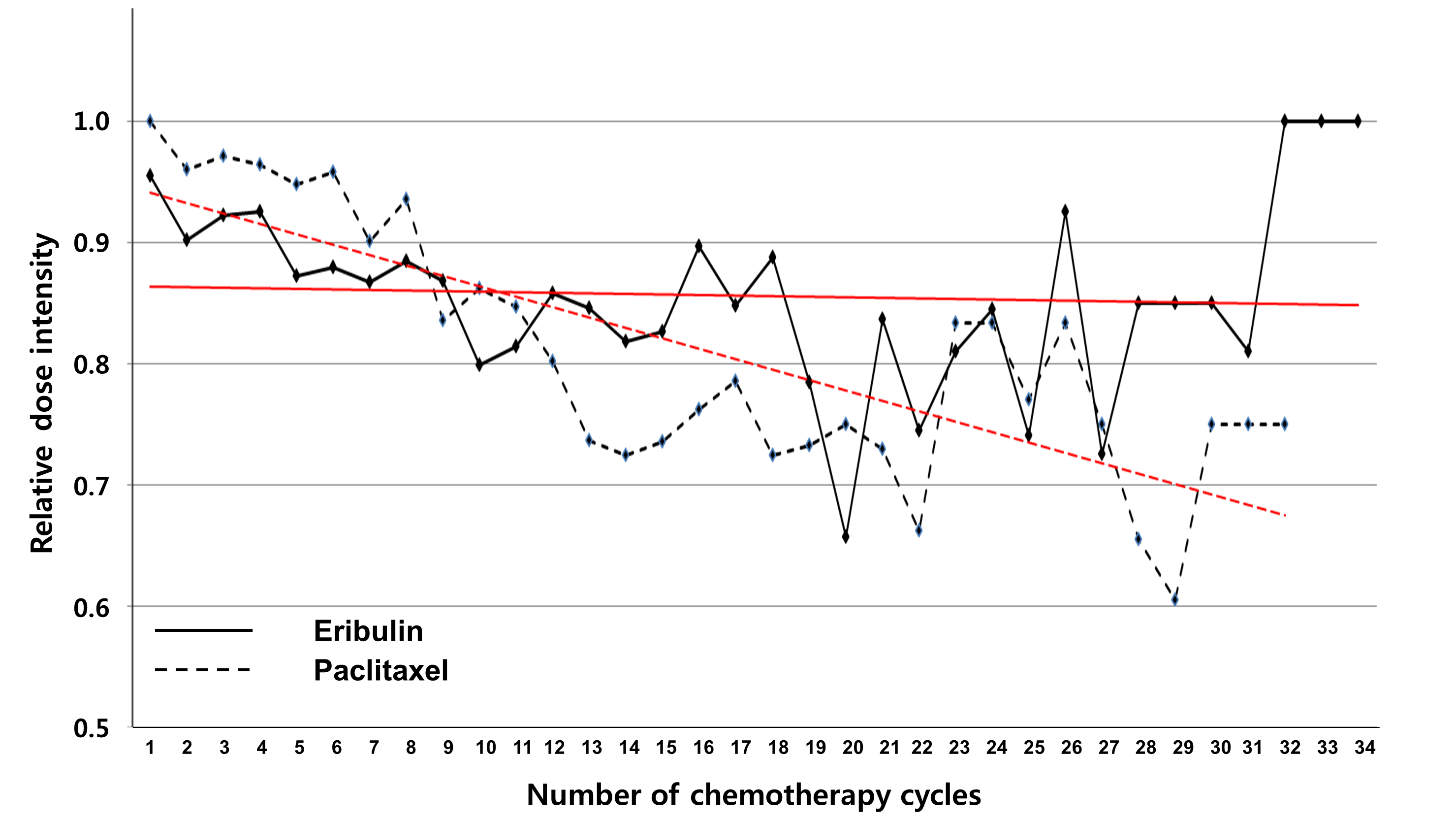

Supplement: Supplementary file 2 — Additional file 2: Figure S1. Relative dose intensities of eribulin and paclitaxel on each cycle. The red lines indicate trends of both arms. [file 40880_2019_375_MOESM2_ESM.tif]
